# Synthetic horsepox viruses and the continuing debate about dual use research

**DOI:** 10.1371/journal.ppat.1007025

**Published:** 2018-10-04

**Authors:** Ryan S. Noyce, David H. Evans

**Affiliations:** 1 Department of Medical Microbiology & Immunology, University of Alberta, Edmonton, Alberta, Canada; 2 Li Ka Shing Institute of Virology, University of Alberta, Edmonton, Alberta, Canada; University of Pittsburgh, UNITED STATES

On January 19, 2018, a paper describing the complete synthesis of a horsepox virus was published in *PLOS ONE* [1]. This is the largest virus assembled to date, and it shows that no viral pathogen is likely beyond the reach of synthetic biology. The paper and reports of its contents have attracted much comment [2–4]. As the two authors who conducted these experiments, we thank *PLOS Pathogens* for giving us the opportunity to address some of the issues arising from this work.

At the heart of the discussion lies the fact that this is dual use research of concern (DURC) [5] because any method that can be used to assemble horsepox virus could be used to construct variola, the virus that causes smallpox. This renders our work of special relevance for the agencies tasked with ensuring that smallpox remains a disease of history. Although the world’s known variola stocks are securely stored in Russia and the United States, synthetic biology compromises this approach for securing any agent. This was shown by the reconstruction of poliovirus in 2002 [6] and has been discussed within WHO [7]. Whether secret or lost [8] stocks of variola virus still exist is unknown, but countermeasures are still stockpiled because of this recognized threat.

If one looks at the opinions that have been expressed about our work, an important point concerns the possibility that it provides instructions for making variola. For good or ill, the world is full of talented scientists who do not need a blueprint to extract knowledge well described in published works [7, 9–11]. This includes papers showing how to assemble a bacmid encoding herpes simplex virus [12] and how to recover vaccinia virus (VACV) from a bacmid [13]. This does not mean this technology is still easily implemented. It is not. All modern “methods” sections omit details familiar to experts. Although variola virus DNA has been recovered from historical specimens [14, 15], it is not accessible by “mail order” [16]. The design work is tricky, 30 kbp plasmids are unstable, and the assembly reactions are inefficient. The skill set needed to do this work requires advanced scientific training, insider knowledge, and infrastructure that is not widely accessible.

Our interest in testing horsepox virus as a potentially safer vaccine was prompted by phylogenetic [17–19] and historical [20–24] evidence suggesting that smallpox vaccines might have originated in horses. Considering the evidence of actual efficacy [22, 24], could a horsepox virus still serve this purpose? Gene synthesis offered a route for obtaining the virus while also providing commercial freedom to operate. But do we still need another smallpox vaccine? There is no short or simple answer to that question, although the Russian and American collaborating centers have reported to WHO that their variola stocks are still being used for ongoing vaccine research [25]. Given this lack of consensus, it seems reasonable to explore other options when considering how best to periodically replenish vaccine stockpiles.

In any discussion of DURC, community risks should be considered alongside community benefits. The risk seems clear, if unquantifiable. What is the benefit? Synthetic biology offers enormous promise as a tool for engineering advanced biotherapeutics. Malaria, HIV, and hepatitis C virus (HCV) remain a challenge from a vaccine perspective, and we are only just beginning to appreciate the complex modifications needed to disarm and retarget poxviruses against cancer (e.g., [26]). For such research to progress, it requires sophisticated tools. Given that approximately 40% of us risk cancer in our lives [27], or the half million who died of malaria last year [28], this promise needs to be considered in balancing risks with potential benefits.

Many comments we have received include complaints that such work “needs to be regulated.” It is implied that Canadian rules were not followed or must be lacking. We would respectfully note that Canada has a long history of thoughtfully managing biosafety and biosecurity issues, and our work was conducted with close attention to stringent safety and security protocols [29]. Canada’s Human Pathogens and Toxins Act [30] is widely viewed as a model for how to manage the risks posed by pathogenic agents, partly because of the consultative way it was implemented [31]. Besides informing WHO of our research interests [7] and obtaining all of the institutional approvals needed to undertake this work, we obtained a legal review of relevant legislation, and the paper was evaluated by four Canadian federal agencies at our request. This thoughtful input guided our preparation of the final document.

So where do we go from here? Realistically all attempts to oppose technological advances have failed over centuries. We suggest that one should instead focus on regulating the products of these technologies while educating people of the need to plan mitigating strategies based upon a sound understanding of the risks that such work might pose. In these discussions, a long-term perspective is essential.

From a regulatory perspective, many countries already control the use of pathogens. That is where the risk resides. Possession of variola virus is a crime in Canada, and other countries have similar laws. Because there are DNA clone libraries [32], WHO recommends that no one should own >20% of the variola genome outside of the two authorized sites [33]. Many countries follow these policies, and some legislate greater restrictions on the size of cloned variola sequences. Therefore, from a biosafety and biosecurity perspective, we already have controls in place to manage the products of these technologies.

**Fig 1:**
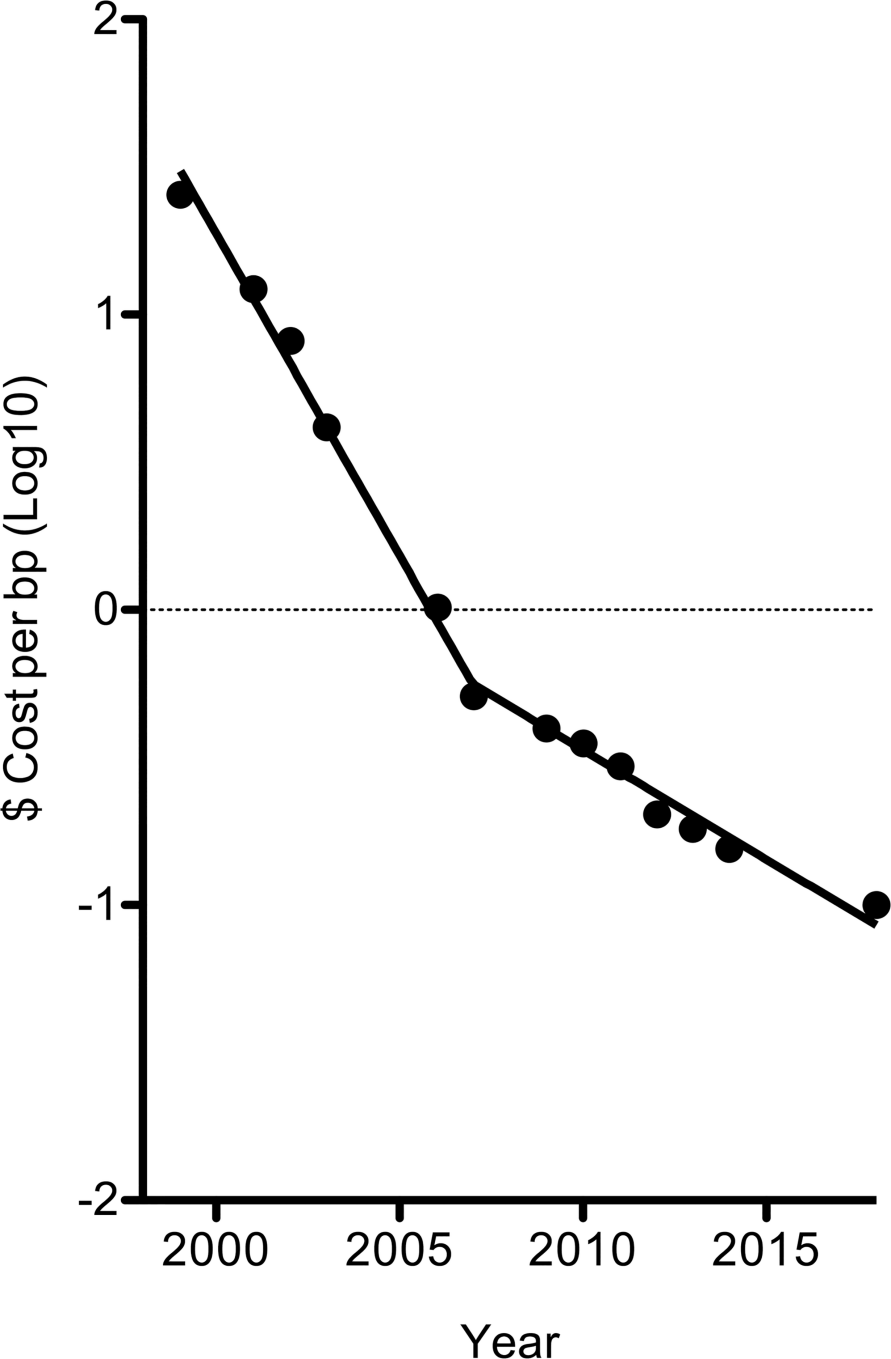
Declining costs of gene synthesis ($US). All of the data are reproduced from [37] except for the latest data point. The cost of assembling large (>10 kbp) DNA clones is generally higher than shown, due to the additional amounts of labor and the quality control that is required.

The bigger challenge concerns education. The “synbio” community has been proactive when considering the implications of their work, as pathogens are just one concern (e.g., [34]). The companies that make DNA screen for similarities to regulated pathogens [35], and this process works well. However, one can now buy kits to perform Gibson assemblies and printers to make the DNA feedstocks. The technology and economics of large-scale DNA synthesis have driven the cost of gene synthesis down approximately 250-fold in just 10 years (Fig 1). WHO’s guidelines relating to variola virus [33] are also unknown to most biologists. We need to figure out how to interdict the materials needed to make synthetic pathogens while educating about the risks posed by these agents even centuries hence.

In conclusion, the authors respect the concerns that have been expressed about this work, but note that our lives have been profoundly improved by technologies, like genetic engineering, that were once viewed as threats to humanity [36]. As the memory of smallpox and polio fades, the challenge will be to educate new generations about the risk posed by these diseases. This necessitates providing the ongoing support that public health agencies will need to protect populations from even “extinct” epidemic diseases. The advance of technology means that no disease-causing organism can forever be eradicated.
